# The contribution of large genomic deletions at the *CDKN2A* locus to the burden of familial melanoma

**DOI:** 10.1038/sj.bjc.6604470

**Published:** 2008-07-08

**Authors:** F Lesueur, M de Lichy, M Barrois, G Durand, J Bombled, M-F Avril, A Chompret, F Boitier, G M Lenoir, French Familial Melanoma Study Group, B Bressac-de Paillerets

**Affiliations:** 1 Groupe Mélanome, Institut Gustave Roussy, FRE2939 CNRS-Université Paris-Sud, Villejuif, France; 2 Service de Génétique, Villejuif, France; 3 Service de Dermatologie, Institut Gustave Roussy, Villejuif, France; 4 AP-HP, Hôpital Cochin, Service de Dermatologie – Université Paris 5, Paris, France; 5 Département de Médecine, Institut Gustave Roussy, Villejuif, France

**Keywords:** melanoma-prone families, *CDKN2A*, p16^INK4a^, p14^ARF^, *KLHL9*, multiplex ligation-dependent probe amplification

## Abstract

Mutations in two genes encoding cell cycle regulatory proteins have been shown to cause familial cutaneous malignant melanoma (CMM). About 20% of melanoma-prone families bear a point mutation in the *CDKN2A* locus at 9p21, which encodes two unrelated proteins, p16^INK4a^ and p14^ARF^. Rare mutations in *CDK4* have also been linked to the disease. Although the *CDKN2A* gene has been shown to be the major melanoma predisposing gene, there remains a significant proportion of melanoma kindreds linked to 9p21 in which germline mutations of *CDKN2A* have not been identified through direct exon sequencing. The purpose of this study was to assess the contribution of large rearrangements in *CDKN2A* to the disease in melanoma-prone families using multiplex ligation-dependent probe amplification. We examined 214 patients from independent pedigrees with at least two CMM cases. All had been tested for *CDKN2A* and *CDK4* point mutation, and 47 were found positive. Among the remaining 167 negative patients, one carried a novel genomic deletion of *CDKN2A* exon 2. Overall, genomic deletions represented 2.1% of total mutations in this series (1 of 48), confirming that they explain a very small proportion of CMM susceptibility. In addition, we excluded a new gene on 9p21, *KLHL9*, as being a major CMM gene.

The incidence of cutaneous malignant melanoma (CMM) (MIM no. 600160) has been increasing rapidly in the Caucasian population in recent decades. Melanoma is a complex and heterogeneous disease with genetic and environmental factors contributing to its development. Population-based studies in Utah and Sweden have reported approximately 2- and 3-fold increased risk of melanoma, respectively, in first-degree relatives of melanoma probands (Goldgar *et al*, 1994; Hemminki *et al*, 2003). Indeed, about 10% of CMM cases occur in a familial setting. Familial melanoma is usually defined as a cluster of two or more first-degree relatives with melanoma. Two high-risk genes have been identified: the cell cycle regulator *CDKN2A* on chromosomal band 9p21 (Hussussian *et al*, 1994) and the *cyclin-dependent kinase-4* (*CDK4*) on chromosomal band 12p14 (Zuo *et al*, 1996), the products of which are known to be components of potent tumour-suppression pathway. The *CDKN2A* gene is the major high-risk CMM susceptibility gene identified to date, as germline mutations in this gene have been found in about 20–40% of melanoma-prone families worldwide (Hayward, 2003). It encodes two distinct proteins, translated in alternate reading frames, from alternatively spliced transcripts. The *α* transcript encodes the p16 inhibitor of cyclin-dependent kinase type 4 protein (p16^INK4a^); the smaller *β* transcript specifies the alternative product p14^ARF^. Both of these proteins are involved in cell cycle regulation. Only two germline mutations in *CDK4* (MIM no. 123829) have been reported worldwide in a total of six *CDKN2A* mutation-negative familial melanoma kindreds, and all mutations occur at the arginine encoded by codon 24 in exon 2 of the gene. This arginine directly interacts with p16, and mutations affecting this codon have the same functional effect as a p16 mutation (Zuo *et al*, 1996; Soufir *et al*, 1998; Molven *et al*, 2005).

Although approximately 50% of melanoma-prone families display linkage to 9p21, only about half of these have been identified as carriers of a mutation in the *CDKN2A* gene (Hussussian *et al*, 1994; Kamb *et al*, 1994; Harland *et al*, 1997; Borg *et al*, 2000; Laud *et al*, 2006). These studies suggest, besides the involvement of *CDKN2A* in susceptibility to melanoma, the possibility of the existence of additional tumour suppressor loci on chromosome 9p21. At the somatic level, the very low frequency of disease-causing alterations (by mutation or inactivation by loss of expression) of the *CDKN2A* gene in sporadic CMM cases with allelic losses of 9p21 is again consistent with the presence of additional tumour suppressor gene(s) within this chromosomal area (Gonzalgo *et al*, 1997; Ruiz *et al*, 1998; Wagner *et al*, 1998). *CDKN2B,* located about 30 kb centromeric from *CDKN2A* and coding the cyclin-dependent kinase inhibitor p15^INK4b^, was the first obvious candidate gene but has been excluded as being a high penetrance susceptibility gene for CMM (Platz *et al*, 1997; Laud *et al*, 2006). The question of whether or not large genomic deletions, undetectable by traditional PCR-amplification and sequencing of individual exons, may be the cause of such unexplained familial clustering of melanoma has been raised, and few authors have searched for large *CDKN2A/ARF* deletions or rearrangements (Fitzgerald *et al*, 1996; Bahuau *et al*, 1998; Randerson-Moor *et al*, 2001; Debniak *et al*, 2004; Mistry *et al*, 2005; Knappskog *et al*, 2006, Erlandson *et al*, 2007). To date, germline large deletions have been characterised at the 9p21 locus in only six families worldwide. A deletion involving *CDKN2A* exon1*α*, 2, and 3 and a deletion removing exon 1*α* and half of exon 2 were described in two melanoma-prone kindreds, originated from UK and from Norway, respectively (Mistry *et al*, 2005; Knappskog *et al*, 2006). Large deletions have also been found in families with combined proneness to melanoma and nervous system tumours (NST): a gross deletion ablating the whole *CDKN2A* and *CDKN2B* genes has been reported in a French family (Bahuau *et al*, 1998; Pasmant *et al*, 2007), and a deletion of p14^ARF^-specific exon 1*β* of the *CDKN2A* gene has been found in one US family and in two UK families (Bahuau *et al*, 1998; Randerson-Moor *et al*, 2001; Mistry *et al*, 2005; Laud *et al*, 2006). A large duplication of the *CDKN2A/CDKN2B* loci has also been reported in a melanoma patient from a Swedish family, but the clinical significance of this variant is not evident (Erlandson *et al*, 2007).

The purpose of the present study was to estimate the prevalence of large deletions at the 9p21 locus, which participated in CMM families originating from France, using the multiplex ligation-dependent probe amplification (MLPA) analysis (Schouten *et al*, 2002). This quantitative technique was used because it enabled fine-scale mapping at the *CDKN2A* locus and allowed other flanking genes to be screened (24 sites of interest on 9p in total). This approach led us to characterise a novel deletion of *CDKN2A* exon 2 (affecting both p14^ARF^ and p16^INK4a^) in one patient. Moreover, it led to the identification of a new variant within the gene *KLHL9* (*Kelch-like 9*) located approximately 630 kb from *CDKN2A* in another patient. Because this variant was not found in a control population, this finding prompted us to investigate this candidate in high-risk melanoma families with no mutation of *CDKN2A*.

## Patients, materials and methods

### Study participants

The patients in this study were enroled through the Department of Dermatology at the Institut de Cancérologie Gustave Roussy and the other French Hospitals that constitute the Familial Melanoma Study Group, as part of the French Familial Melanoma Project (Grange *et al*, 1995). A total of 214 index cases from independent pedigrees with at least two melanoma cases were identified between 1986 and 2005. These cases had confirmed diagnosis of CMM, through medical records, review of pathological material, and/or pathological reports. Among the 214 index cases, direct sequencing of *CDKN2A* (exon 1*β*, 1*α*, 2 and 3) or *CDK4* (exon 2) had identified *CDKN2A* point mutations or small insertions/deletions in 46 families, and the *CDK4* codon 24 point mutation in one family (Supplementary Table 1). The remaining 167 index cases were included in the present study to evaluate the contribution of large genomic deletions at 9p21 in the French series. Probands from these negative families displayed the following inclusion criteria: a) families with at least three affected members (‘high-risk family set’, *N*=42), (b) families with two first-degree relatives affected with confirmed melanoma (*N*=102), (c) families with two second degree relatives affected with confirmed melanoma (*N*=23). The mutation scanning of *KLHL9* was performed in patients from the high-risk family set, including the 42 index cases and 48 additional affected members. Our control population consisted of 188 DNA samples prepared from lymphocytes of blood donors born in France. The study was approved by an institutional review board-approved protocol (CCPPRB no: 01-09-05, Paris Necker). It was conducted according to the Declaration of Helsinki Principles. All participating subjects signed informed consent before providing blood samples. DNA from participants was extracted from peripheral blood lymphocytes, using the QIAamp DNA Blood mini kit (QIAGEN, Hilden, Germany), according to the manufacturer's guidelines.

### Large insertion and deletion analysis

Deletion screening of the *CDKN2A* locus was carried out using the 9p21 Multiplex ligation-dependent probe amplification kit (P024), according to the supplied protocol (MRC-Holland, Amsterdam, the Netherlands) (Schouten *et al*, 2002; Mistry *et al*, 2005). The technique and preparation of the probes used in this kit are described elsewhere (Schouten *et al*, 2002; Mistry *et al*, 2005). Gene dosage quotients for 9 *CDKN2A* locus sites and for 15 other 9p genes sites were determined to screen for deletions at 9p. In total, 21 specific probes mapped to 9p21 (three within *CDKN2B*, three within *MTAP*, one within *IFNA1*, one within *KIAA1354* (*KLHL9*), one within *IFNW1*, one within *IFNB1*, one within *ELAVL2*, one within *TEK*), two probes mapped to 9p22 (both within *MLLT3*) and one probe mapped to 9p24 (within *FLJ00026 (DOCK8*)).

Calculation of dosage quotients was carried out as described by Mistry *et al* (2005). Theoretically, gene dosage quotients close to 1.0 indicate two copies present (that is, wild-type); 0.5, one copy absent (that is, hemizygous); 0.0, both copies absent (that is, homozygous deletion); and 1.5, one copy duplicated. Quotients were scored according to observations made from known samples: wild type if the quotient was between 0.8 and 1.2, hemizygous deletion if the quotient was between 0.4 and 0.7, homozygous deletion if the quotient was between 0.0 and 0.2. All other values were considered undetermined.

### RT–PCR analysis of *CDKN2A* transcripts

RNA from patient carrying the heterozygous *CDKN2A* deletion was extracted from peripheral blood lymphocytes using the AllPrep DNA/RNA Mini Kit (QIAGEN), and cDNA was synthesized by reverse transcription using the Superscript reverse transcriptase system (Invitrogen, Paisley, UK) according to the manufacturer's recommendations.

### Characterisation of *CDKN2A* deletion breakpoint

Long-range PCRs were performed with the Expand™ Long Template PCR System (Boehringer Mannheim, Mannheim, Germany) as recommended by the manufacturer. Primers were designed to amplify the region encompassing the putative deletion between exon 1*α* and exon 3. Forward primer and reverse primer amplified a 355 bp mutant p16^INK4^-encoding cDNA fragment lacking exon 2, the wild type cDNA fragment being 662 bp long (Table 1). To characterise the intronic deletion breakpoints, forward primer in exon 1*α* and reverse primer in intron 2 amplified a 3705 bp genomic fragment for the wild type allele and a 770 bp fragment for the mutant allele (Table 1).

PCR products were sequenced with the Big Dye Terminator, version 3.0 (Applied Biosystems, Foster City, CA, USA) on the ABI Prism^©^ 3730 Genetic Analyzer (Applied Biosystems, Foster City, CA, USA).

### Direct sequencing of *KLHL9*

Primers used for the sequencing of *KLHL9* are given in Table 1. The amplification protocol consisted of 35 cycles with temperature steps at 94, 60, and 72°C for 30 s each.

## Results

### Contribution of genomic rearrangements at 9p21

**Figure 1:**
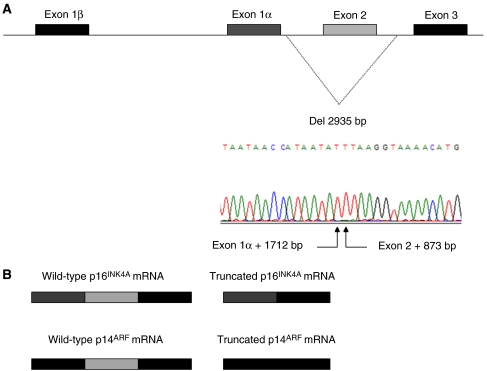
Characterization of the new *CDKN2A* deletion. (**A**) Genemap of the *CDKN2A* locus. Breakpoints of the deletion (exon 1*α* +1712bp_exon 2 +873 bp) are indicated and illustrated by the sequence chromatogram. (**B**) Comparison of the wild type p16^INK4A^ and p14^ARF^ transcripts arising from the allele harbouring the deletion. The truncated transcripts are lacking exon 2. This alters the reading frame of both transcripts.

Among the 167 index cases screened for genomic rearrangements at 9p21, 166 patients present a gene dosage quotient close to one (between 0.8 and 1.2), indicating no alteration in gene copy numbers, and one patient (0.6%) was identified to carry a hemizygous deletion of *CDKN2A* exon 2, as gene dosage quotient was close to 0.5. Multiplex ligation-dependent probe amplification probes at exon 1*β*, exon1*α* and exon 3 of *CDKN2A* did not reveal a reduced gene dosage quotient for this patient, indicating that, according to position of probes within the exons, the deletion was at most 6.4 kb long with intronic breakpoints, extending at most from the first base of intron 1*α* to the last base of intron 2 (Figure 1A). These results were confirmed by quantitative multiplex PCR of short fluorescent fragments with primers specifically amplifying *CDKN2A* exon 1*β*, exon1*α*, exon 2 and exon 3 (data not shown) (Casilli *et al*, 2002). To verify whether or not the putative *CDKN2A* mutation generated aberrant mRNAs, long range PCR was performed on the patient cDNA. Sequencing of the PCR product confirmed that the germline deletion leads to an aberrant p16^INK4a^ transcript, lacking the 307 bp corresponding to *CDKN2A* exon 2 (Figure 1). To determine the exact nature of the *CDKN2A* gene aberration, long range PCR and sequencing was performed on the patient's genomic DNA. This revealed a 2935 bp deletion extending from nucleotide position +1712 bp from end of exon 1*α* to position +873 bp from end of exon 2 (Figure 1). It is worth noting that the deletion breakpoints did not lie within ALU sequences, and that such repetitive elements could not explain the occurrence of a rearrangement at this genomic position.

**Figure 2:**
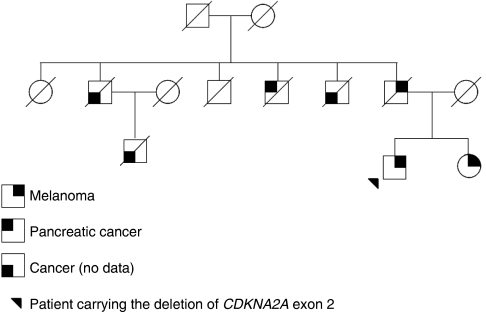
Family tree of patient carrying the *CDKN2A* exon 2 deletion.

The patient carrying this novel deletion of *CDKN2A* presented with dysplastic nevus syndrome and developed five primary melanomas between 45 and 51 years of age. He was the index case of a melanoma-prone family of three affected members: his father had a confirmed diagnosis of melanoma at the age of 50, and his sister was also reported to have had melanoma, but no pathological report was available for her (Figure 2). We were unable to investigate the co-segregation of the genomic deletion with melanoma in this pedigree because of unavailability of biological material for the index case's relatives. In addition, the patient's uncle died of pancreatic cancer, a cancer that had been associated with *CDKN2A* germline mutations (Goldstein *et al*, 1995; Goldstein *et al*, 2006), and three other family members died of cancer, but no clinical details were reported to the clinician (Figure 2).

### *KLHL9* on 9p21 as a new candidate gene for susceptibility to melanoma

**Figure 3:**
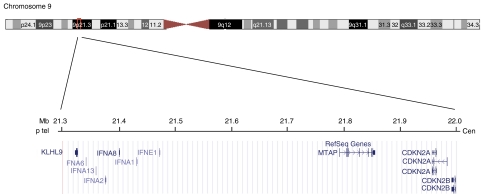
Overview of the 9p21 region containing *KLHL9* and *CDKN2A* locus.

**Figure 4:**
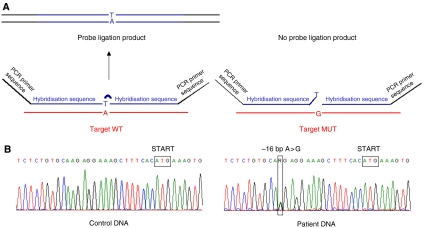
Identification of *KLHL9* variant. (**A**) Principle of MLPA analysis: the patient carrying the variant shows a reduced amount of amplification product for probes specific of the promoter region of *KLHL9.* (**B**) Sequence chromatograms illustrating the nucleotidic change A>G at position –16 of the gene that prevents the ligation reaction.

**Figure 5:**
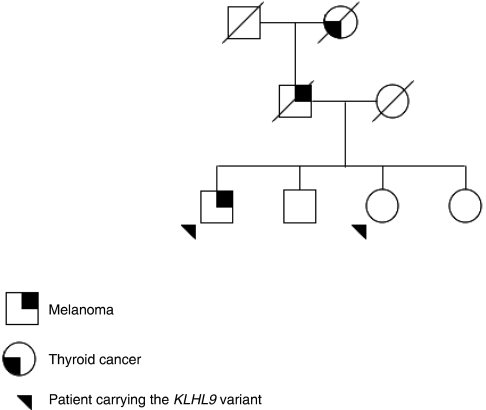
Family tree of patient carrying the *KLHL9* variant.

For a second patient diagnosed with melanoma at the age of 54, and belonging to a different melanoma -prone pedigree, a dosage quotient close to 0.5 was obtained for a 9p probe specific of the 5′ end of the transcription unit of the single exon gene *KLHL9* (Kelch-like 9, previously named *KIAA1354*), suggesting a hemizygous deletion in this gene located about 630 kb telomeric from *CDKN2A* (Figure 3). Subsequent direct sequencing of the entire exon led to the identification of a heterozygous nucleotidic variation (A>G) located 16 bp upstream of the START codon of *KLHL9* (NM_018847), that prevents the ligation reaction of the two contiguous probes used for MLPA, for the G allele (Figure 4). Although the various *in silico* tools (PupaSuite, http://pupasuite.bioinfo.cipf.es/, CorePromoter,http://rulai.cshl.org/tools/genefinder/CPROMOTER/, TFSEARCH, http://www.cbrc.jp/research/db/TFSEARCH.html, TESS, http://www.cbil.upenn.edu/cgi-bin/tess/tess) did not suggest that the presence of the G allele would have direct functional effects on the activity of the promoter, this new variant was not described in the public SNPs databases (ENSEMBL, http://www.ensembl.org/, dbSNP, http://www.ncbi.nlm.nih.gov/SNP, and HAPMAP, http://www.hapmap.org/) and was absent in 188 unrelated controls from France (376 chromosomes). DNA was available for the two unaffected siblings of the patient, and MLPA and sequence analysis revealed that one of the patient's sisters, unaffected at age 62, also carried the –16 A>G allele. The father of the patient died of melanoma metastases to the brain at the age of 70, and his paternal grandmother had a suspicious pigmented lesion on her face and died of thyroid cancer at the age of 80. Unfortunately, material was not available for any of them, and co-segregation of the variant with skin lesion could not be investigated (Figure 5).

Beside its genomic location at 9p21, *KLHL9* appeared to represent a good candidate for melanoma susceptibility as it has been reported that loss of function of BTB/kelch repeat proteins may contribute to tumorigenesis (Adams *et al*, 2000; Liang *et al*, 2004; Yoshida, 2005). Therefore, we undertook the resequencing of the 4.3 kb unique exon of the gene in 90 CMM patients belonging to the high-risk family set (that is, families counting at least 3 CMM) with no *CDKN2A* or *CDK4* mutation. The variant identified in the promoter of *KLHL9* was not found in this series, and no other mutation elsewhere in the candidate gene was detected by direct sequencing.

## Discussion

By adding a novel germline large deletion to the repertoire of germline *CDKN2A* mutations, our data contribute to an enlargement of the spectrum of mutations identified in melanoma-prone families and improve the estimate of the prevalence of this type of lesion, as the work was performed on the largest family dataset to date. Among the 214 melanoma-prone families recruited through the French Familial Melanoma Project, 46 families were positive for point mutations within *CDKN2A* (21.5%), one family carried a *CDK4* point mutation in exon 2 (0.5%), and one family carried a large *CDKN2A* deletion (0.5%). Overall, a genetic alteration affecting either *CDKN2A* or *CDK4* was detected in 48 of 214 (22.4%) of the French CMM families. Point mutations account for 97.9% (47 of 48), and large genomic rearrangements account for 2.1% (1 of 48) of *CDKN2A*/*CDK4* mutations. The frequency of such alterations detectable by MLPA in CMM families negative for point mutations in *CDKN2A* and *CDK4* (0.6%; 1 of 167 pedigrees screened) is even lower than frequencies reported in the UK population, 3.2% (3 of 93 pedigrees screened) (Mistry *et al*, 2005). Therefore, the question of systematic MLPA screening in pedigrees with no point mutations in the coding sequence of *CDKN2A* could be debated, as such screening will not improve substantially the sensitivity of genetic testing.

Owing to the overlapping open reading frames in exon 2, this deletion of *CDKN2A* is a new example of a germline mutation affecting p16 and p14 proteins, two major regulators of cell cycle progression and survival through the pRb and p53 pathways, respectively. The functional relevance of this genomic deletion is inferred based on three observations: (1) the knowledge that p16 proteins with less than 120 residues lack the capacity to bind to and inhibit CDK (Parry and Peters, 1996, Hashemi *et al*, 2000, 2002), (2) loss of *CDKN2A* exon 2 has been shown to reduce the activity of p14 (Hashemi *et al*, 2000, 2002), (3) both p14 and p16 mRNA lacking exon 2 are translated into truncated proteins when expressed exogenously (Hashemi *et al*, 2000). In addition, it is also possible that the *CDKN2A* exon 2 deletion leads to aberrant transcripts, which are the target of the nonsense-mediated mRNA decay mechanism, thus preventing the synthesis of truncated p14 and p16.

Interestingly, three other gross deletions at the *CDKN2A* locus, impacting on both p16 and p14, have been reported from melanoma-NST families (Bahuau *et al*, 1998; Randerson-Moor *et al*, 2001; Mistry *et al*, 2005; Pasmant *et al*, 2007), and a splice site mutation removing *CDKN2A* exon 2 was also found in a melanoma/neurofibroma family (Petronzelli *et al*, 2001). However, there was no evidence of NST in the family with the novel *CDKN2A* deletion presented here, although clinical description of this family was very basic.

Methods used to screen for mutations in *CDKN2A/ARF* are usually PCR-based and focus on the detection of small sequences alterations, such as point mutations, small deletions and insertions. Few investigators have systematically searched for large *CDKN2A/ARF* deletions or rearrangements (Fitzgerald *et al*, 1996; Debniak *et al*, 2004; Mistry *et al*, 2005). We chose to apply MLPA analysis to determine whether large deletions or rearrangements occur at the *CDKN2A/ARF* locus on 9p21 in French CMM families; however, the failure to detect large deletions using this technique does not exclude the possibility that large deletions may exist, as the probes used do not cover the entire genomic sequence of *CDKN2A*. Moreover, neither inversions, nor translocations are detectable by this approach, and such genomic alterations cannot be ruled out in melanoma families where no *CDKN2A* mutation has been detected. Furthermore, the possibility of alteration at the transcript level in high-risk families with no mutation identified through conventional testing should be considered (Knappskog *et al*, 2006). As we did not employ RT–PCR, deep intronic mutations that have the potential to alter intron/exon splicing could have been missed. Finally, absence of *CDKN2A* mutation or large deletion does not rule out the possibility that epigenetic modification of either p16 or p14*ARF* could result in gene silencing that would contribute to melanoma (Merlo *et al*, 1995; Esteller *et al*, 2000). Such a germline epimutation silencing the DNA mismatch repair gene *MLH1* has been reported in nonpolyposis colorectal cancer (Suter *et al*, 2004; Hitchins *et al*, 2007).

In conclusion, although approximately 50% of melanoma-prone families display linkage to 9p21, only about half of these have been identified as carriers of mutations in the *CDKN2A* gene by direct exon sequencing (Hussussian *et al*, 1994; Kamb *et al*, 1994; Harland *et al*, 1997; Borg *et al*, 2000). Few of the melanoma-prone families tested here were sufficiently informative to assess for linkage to 9p21. However, the present work provides evidence that only a small fraction of the unexplained familial risk of CMM is attributable to large deletions of *CDKN2A* locus. Therefore, there may be yet unidentified highly penetrant melanoma susceptibility genes located at the 9p21 locus. The finding of the 5′UTR variant within *KLHL9*, located 630 kb from *CDKN2A* on 9p21 in one melanoma patient was serendipitous. Little is known about the biological function of *KLHL9* itself, but Kelch-superfamily proteins participate in various cellular activities, and functions of family members impinge on cell morphology, cell organisation and gene expression. *KLHL* (kelch homologue) genes contain two evolutionary conserved domains: a broad-complex, tramtrack, bric-à-brac/proxvirus and zinc finger (BTB/POZ) domain, and a kelch motif, which is a human homologue of the Drosophila *kelch* gene. Kelch-like proteins are much conserved, and some family members have been implicated in embryogenesis and carcinogenesis through cytoskeleton organisation (Adams *et al*, 2000; Liang *et al*, 2004; Yoshida, 2005). Thus, as for other BTB/kelch repeat proteins, we hypothesized that loss of function of KLHL9 may contribute to tumorigenesis by promoting cell proliferation, suppressing apoptosis, and/or by affecting nuclear cytoskeleton dynamics. Although the 5′UTR variant within *KLHL9* was absent in a French control population, we ruled out major involvement of *KLHL9* in the susceptibility to melanoma by screening the entire coding sequence of the candidate gene in a panel of 42 high-risk CMM families. Recently, a new large antisense non coding RNA, named *ANRIL*, has been identified at the *CDKN2A* locus, with a first exon located in the promoter of the *p14/ARF* gene and overlapping the two exons of *p15/CDKN2B* (Pasmant *et al*, 2007). As *ANRIL* was localised within the 403 kb deletion in the French melanoma-NST family (Bahuau *et al*, 1998), it has been hypothesized that this new gene could be involved melanoma-NST syndrome families and in melanoma-prone families with no identified *CDKN2A* mutations. The possible involvement of *ANRIL* has not been investigated yet, and further studies should elucidate the role of this gene in the susceptibility to CMM.

Finally, efforts are currently underway to identify new high penetrance susceptibility genes on other chromosomes, as there remain a significant proportion of CMM families (∼50%) that are not linked to chromosome 9p21. Such genes may lie at 1p36 (Goldstein *et al*, 1993) and 1p22 (Gillanders *et al*, 2003).

## Figures and Tables

**Table 1:** Primer sequences for characterization of *CDKN2A* exon 2 deletion and screening of the *KLHL9* gene

**Gene**	**Amplicon**	**Forward sequence 5′ >3′**	**Reverse sequences 5′>3′**
*CDKN2A*	Exon 1*α*–Exon 3	CGCCAGCACCGGAGGAAGAA	CCTGTAGGACCTTCGGTGACTGA
	Exon 1*α*–Intron 2	TTTTCTTTTTGCTTTGGATTTCTA	AAGGGAGGAGGGAAGAAATGA
*KLHL9*	Amplicon 1	GCTGACTGACGAGGTCTGG	GAGATGCAAGCCGGATAAAG
	Amplicon 2	TGGTCCTGCTACAGGGTGAT	GCAATTCGTCCAACCTCAAC
	Amplicon 3	TGGACAATCTTCAGGACACACT	CTGCTGCCTCAAAACTCCTC
	Amplicon 4	ACCTGCGTGAATTTGCTTTT	CATGGGTAATTCCTCCTGAAA
	Amplicon 5	CTGGTGAACTGGCCACAGTA	GTGACCCAGGGTTTTCTTCA
	Amplicon 6	GACCCAGAAAAAGATGAGTGG	AATGCAACCACACAATCGAG
	Amplicon 7	GTTGAGTTTTCATCTTTGACTCCA	TGCACAAAAGCAGTTCTCTGA
	Amplicon 8	AAATCGACAACCAAATTTGTCA	TGTGGTTCTCAACCAATAGGG
	Amplicon 9	ATTCCCAAAGAAGCCAGCTA	GGATGCACTTCTTGCTTATTCA
	Amplicon 10	GCATTGGCAGAAATTTTCATACT	CACCTTGATATGTCAGAATAAGCAC
