# Supplementary material for: The contribution of large genomic deletions at the CDKN2A locus to the burden of familial melanoma
Source: Br J Cancer. 2008 Jul 8;99(2):364–70. doi: 10.1038/sj.bjc.6604470 (PMC2480975; doi:10.1038/sj.bjc.6604470)
Supplement: Supplementary Table 1 [file 6604470x1.doc]

**Supplementary Table 1.** *CDKN2A* and *CDK4* point mutations, small deletions or insertions identified by direct sequencing in the 47 French families with at least 2 confirmed melanoma cases

| Family ID | Reference | Gene | Location | Nucleotide change | Amino acid change |
| --- | --- | --- | --- | --- | --- |
| 11629 | This article | *CDKN2A* | 5'UTR | -34G>T | - |
| 12581 | This article | *CDKN2A* | 5'UTR | -34G>T | - |
| 13854 | This article | *CDKN2A* | 5'UTR | -34G>T | - |
| 12649 | This article | *CDKN2A* | 5'UTR | -34G>T | - |
| 14396 | This article | *CDKN2A* | 5'UTR | -34G>T | - |
| 1115 | Soufir *et al,* 1998; Chaudru *et al,* 2004; Goldstein *et al,* 2006 | *CDKN2A* | Exon 1α | 71G>C | Arg24Pro |
| 18146 | This article | *CDKN2A* | Exon 1α | 71G>C | Arg24Pro |
| 3180 | This article | *CDKN2A* | Exon 1α | 73delG | Val25X |
| 18160 | This article | *CDKN2A* | Exon 1α | 142C>A | Pro48Thr |
| 13827 | This article | *CDKN2A* | Exon 1α | 146T>G | Ile49Ser |
| 11753 | This article | *CDKN2A* | Intron 1 | 151-2A>G | - |
| 1463 | Soufir *et al,* 1998; Chaudru *et al,* 2004 | *CDKN2A* | Exon 2 | 159G>C | Met53Ile |
| 2722 | This article | *CDKN2A* | Exon 2 | 159G>C | Met53Ile |
| 1352 | Soufir *et al,* 1998; Chaudru *et al,* 2004; Kannengiesser *et al,* 2007 | *CDKN2A* | Exon 2 | 167G>T | Ser56Ile |
| 1468 | Soufir et al, 1998; Kannengiesser et al, 2008 | *CDKN2A* | Exon 2 | 170C>T | Ala57Val |
| 251 | Soufir *et al,* 1998; Yakobson *et al,* 2003; Chaudru *et al,* 2004; Goldstein *et al,* 2006 | *CDKN2A* | Exon 2 | 176T>G | Val59Gly |
| 19024 | This article | *CDKN2A* | Exon 2 | 176T>G | Val59Gly |
| 15149 | Kannengiesser *et al,* 2008 | *CDKN2A* | Exon 2 | 178_179delinsCG | Ala60Arg |
| 11384 | Kannengiesser *et al,* 2008 | *CDKN2A* | Exon 2 | 192_194dup | Leu65dup |
| 3359 | Kannengiesser *et al,* 2008 | *CDKN2A* | Exon 2 | 199G>C | Gly67Arg |
| 2729 | Chaudru *et al,* 2004; Kannengiesser *et al,* 2008 | *CDKN2A* | Exon 2 | 199_213del | Gly67_Asn71del |
| 10381 | This article | *CDKN2A* | Exon 2 | 212A>G | Asn71Ser |
| 10704 | Kannengiesser *et al,* 2008 | *CDKN2A* | Exon 2 | 220G>T | Asp74Tyr |
| 12273 | This article | *CDKN2A* | Exon 2 | 250G>A | Asp84Asn |
| 265 | Kannengiesser *et al,* 2008 | *CDKN2A* | Exon 2 | 259C>T | Arg87Trp |
| 894 | Soufir *et al,* 1998, Goldstein *et al,* 2006; Kannengiesser *et al,* 2008 | *CDKN2A* | Exon 2 | 296G>C | Arg99Pro |
| 2548 | Soufir *et al,* 1998; Chaudru *et al,* 2004; Goldstein *et al,* 2007 | *CDKN2A* | Exon 2 | 301G>T | Gly101Trp |
| 10445 | This article | *CDKN2A* | Exon 2 | 301G>T | Gly101Trp |
| 1220 | Soufir *et al,* 1998; Chaudru *et al,* 2004; Goldstein *et al,* 2007 | *CDKN2A* | Exon 2 | 301G>T | Gly101Trp |
| 1379 | Soufir *et al*, 1998; Ciotti *et al*, 2000; Chaudru *et al,* 2004; Goldstein *et al*, 2006; Goldstein *et al*, 2007 | *CDKN2A* | Exon 2 | 301G>T | Gly101Trp |
| 12193 | This article | *CDKN2A* | Exon 2 | 301G>T | Gly101Trp |
| 14467 | This article | *CDKN2A* | Exon 2 | 301G>T | Gly101Trp |
| 14682 | This article | *CDKN2A* | Exon 2 | 301G>T | Gly101Trp |
| 18690 | This article | *CDKN2A* | Exon 2 | 301G>T | Gly101Trp |
| 9988 | This article | *CDKN2A* | Exon 2 | 305_308del | Ala102fs |
| 11758 | This article | *CDKN2A* | Exon 2 | 334C>G | Arg112Gly |
| 15505 | This article | *CDKN2A* | Exon 2 | 339_340delinsCT | Pro114Ser |
| 15750 | This article | *CDKN2A* | Exon 2 | 339_340delinsCT | Pro114Ser |
| 15106 | This article | *CDKN2A* | Exon 2 | 340C>T | Pro114Ser |
| 12082 | This article | *CDKN2A* | Exon 2 | 377T>A | Val126Asp |
| 2899 | This article | *CDKN2A* | Exon 2 | 377T>A | Val126Asp |
| 18730 | This article | *CDKN2A* | Intron 2 | 458-105A>G | - |
| 17267 | This article | *CDKN2A* | Intron 2 | 458-105A>G | - |
| 11042 | This article | *CDKN2A* | Intron 2 | 458-105A>G | - |
| 9877 | This article | *CDKN2A* | Intron 2 | 458-2A>C | - |
| 14913 | This article | *CDKN2A* | Intron 2 | 458-2A>C | - |
| 759 | Soufir *et al,* 1998 | *CDK4* | Exon 2 | 70G>A | Arg24His |
